# Malignant phyllodes tumor in the right breast and invasive lobular carcinoma within fibroadenoma in the other: case report

**DOI:** 10.1590/S1516-31802000000200004

**Published:** 2000-03-02

**Authors:** Luiz Henrique Gebrim, Júlio Roberto de Macedo Bernardes, Afonso Celso Pinto Nazário, Cláudio Kemp, Geraldo Rodrigues de Lima

**Keywords:** Fibroadenoma, Phyllodes Tumor, Breast Cancer, Fibroadenoma, Tumor Phyllodes, Câncer de Mama

## Abstract

**CONTEXT:**

The malignant variety of the phyllodes tumor is rare. The occurrence of invasive lobular carcinoma within fibroadenoma is rare as well.

**DESIGN:**

Case report.

**CASE REPORT:**

A 58-year-old black female patient was referred to the Mastology unit of the Department of Gynecology, Federal University of São Paulo / Escola Paulista de Medicina, in February 1990, presenting an ulcerated tumor in the right breast with fast growth over the preceding six months. She was a virgin, with menopause at the age of 45 years and had not undergone hormone replacement treatment. The physical examination showed, in her right breast, an ulcerated tumor of 20 × 30 cm which was not adherent to the muscle level, multilobular and with fibroelastic consistency. The axillary lymph nodes were not palpable. The left breast showed a 2 × 3 cm painless, movable nodule, with well-defined edges, and fibroelastic consistency. We performed left-breast mammography, which showed several nodules with well-defined edges, the largest being 2 × 3 cm and exhibiting rough calcification and grouped microcalcifications within it. The patient underwent a frozen biopsy that showed a malignant variant of the phyllodes tumor in the right breast and fibroadenoma in the left one. After that, we performed a total mastectomy in the right breast and an excision biopsy in the left one. Paraffin study confirmed the frozen biopsy result from the right breast, yet we observed that in the interior of the fibroadenoma that was removed on the left, there was a focal area of invasive lobular carcinoma measuring 0.4 cm. The patient then underwent a modified radical mastectomy with total axillary lymphadenectomy. None of the 21 dissected lymph nodes showed evidence of metastasis. In the follow-up, the patient evolved asymptomatically and with normal physical and laboratory examination results up to July 1997.

## resumo

**CONTEXTO:**

O tumor Phyllodes variedade maligna é raro na literatura. A associação entre fibroadenoma e carcinoma lobular invasivo no seu interior também é rara.

**OBJETIVO:**

Descrever a concomitância das três neoplasias na mesma paciente, situação esta ainda não descrita.

**TIPO DE ESTUDO:**

Relato de caso.

**RELATO DE CASO:**

Paciente de 58 anos, mulher, foi atendida na Unidade de Mastologia do Departamento de Ginecologia da Universidade Federal de São Paulo em fevereiro de 1990, apresentando tumor ulcerado na mama direita com rápido crescimento nos últimos seis meses. Era virgem, com menopausa aos 45 anos e não recebeu reposição hormonal. Ao exame físico, tumor ulcerado na mama direita, de 20×30 cm que não estava aderida aos planos musculares, era multilobular e com a consciência fibroelástica. Linfonodos axilares não eram palpáveis. Na mama esquerda, um nódulo móvel de 2×3 cm, com limites bem definidos e consistência fibro-elástica. Foi realizada mamografia na mama esquerda que mostrou vários nódulos de limites bem definidos. O maior de 2×3 cm exibindo calcificação e um grupo de microcalcificações. A biópsia por congelação apresentou uma variação maligna do tumor de phyllodes na mama direita e fibroadenoma na esquerda. Foi realizada a mastectomia direita e biopsia excisional esquerda. O exame histopatológico confirmou o resultado da biópsia por congelação da mama direita e apresentou no interior da fibroadenoma da mama esquerda uma área apenas com carcinoma invasivo lobular medindo 0.4 cm. A paciente foi submetida a mastectomia radical com linfadenectomia axilar total. Nenhum dos 21 nódulos dissecados apresentou metástase. No seguimento, a paciente evoluiu assintomática até julho de 1997.

## INTRODUCTION

Fibroadenoma is the most frequent benign neoplasia of the breast, but the malignant transformation of its epithelial component is rare. In fact, it occurs in only 0.02 to 0.1% of cases.^1^ The transformation of fibroadenoma into a phyllodes tumor is still controversial. The latter is a rare fibroepithelial neoplasia which corresponds to 0.5% of the reported breast tumors. Its biological behavior is distinct from that of fibroadenoma, since it shows between 20 and 40% of local recurrence, and if malignant may cause metastasis.^2^

Until now, the concomitance of an invasive carcinoma within fibroadenoma and a malignant phyllodes tumor in the same patient had never been reported. This is why the present case is now being reported.

## CASE REPORT

A 58-year-old black female patient was referred to the Mastology unit of the Department of Gynecology, Federal University of São Paulo / Escola Paulista de Medicina, in February 1990, presenting an ulcerated tumor in the right breast with fast growth over the preceding six months. She was virgin, with menopause at the age of 45 years and had not undergone hormone replacement treatment.

**Fig 1:**
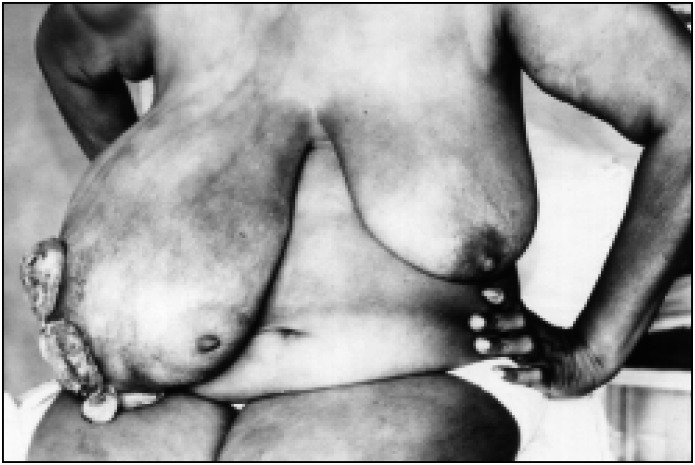
Bulky ulcerated tumor in the right breast.

The physical examination showed, in her right breast, an ulcerated tumor of 20 × 30 cm which was not adherent to the muscle level, multilobular and with fibroelastic consistency (Figure 1). The axillary lymph nodes were not palpable. The left breast showed a 2 × 3 cm painless, movable nodule, with well-defined edges, and fibroelastic consistency.

**Fig 2:**
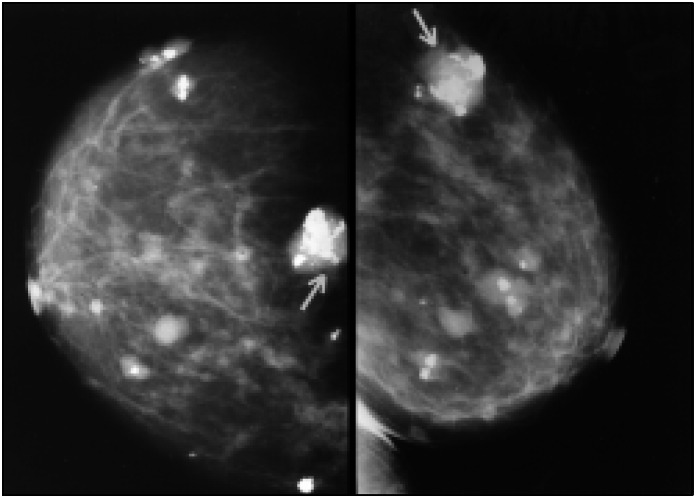
Mammography of the left breast showing several lobular nodules with rough calcifications within most of them, the largest showing grouped and heterogeneous microcalcifications (arrow).

We performed left-breast mammography, which showed several nodules with well-defined edges, the largest being 2 × 3 cm and exhibiting rough calcification and grouped microcalcifications within it (Figure 2).

The patient underwent a frozen biopsy that showed a malignant variant of the phyllodes tumor in the right breast and fibroadenoma in the left one.

After that, we performed a total mastectomy in the right breast and an excision biopsy in the left one.

**Fig 3:**
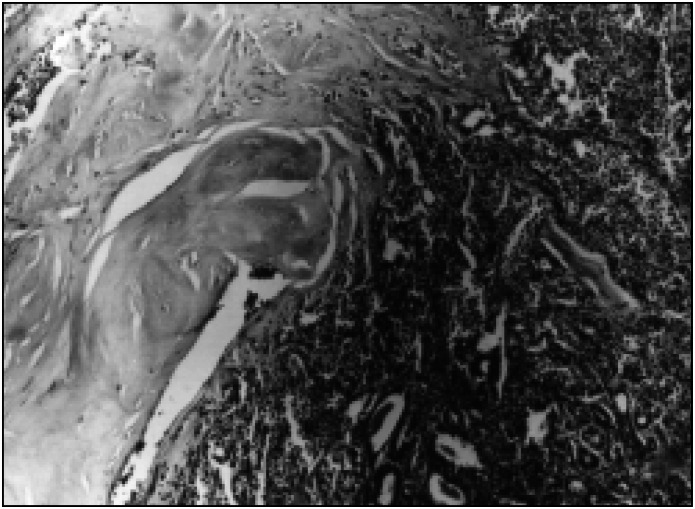
Photomicrograph (hematoxilin & eosin – 100X) showing an area of invasive lobular carcinoma within a fibroadenoma.

Paraffin study confirmed the frozen biopsy result from the right breast, yet we observed that in the interior of the fibroadenoma that was removed on the left, there was a focal area of invasive lobular carcinoma measuring 0.4 cm (Figure 3). The patient then underwent a modified radical mastectomy with total axillary lymphadenectomy. None of the 21 dissected lymph nodes showed evidence of metastasis. In the follow-up, the patient evolved asymptomatically and with normal physical and laboratory examination results up to July 1997.

## DISCUSSION

Fibroadenoma is a frequent cause of nodules in young women, with a peak incidence between 20 and 30 years of age. They are multiple in 15% of the patients. They originate from the breast lobules and are estrogen-dependent, as they grow during pregnancy and under hormone replacement therapy, participate in lactation and often decrease in the menopause.^1,2^

Fibroadenoma is not associated with an increased risk of breast cancer. However, DuPont et al.^3^ observed in a case-control study that complex fibroadenomas (those with cysts greater than 3 mm in diameter, sclerosing adenosis, epithelial calcifications or papillary changes) increased the relative risk for breast cancer to 3.1.

It is known that more than 160 cases of carcinomas originating from fibroadenomas have been reported. Most of the lesions have been characterized as lobular carcinomas *in situ*. Intraduct carcinoma was identified in 20% of the cases, invasive duct carcinoma in 20%, and invasive lobular carcinoma in 10%.^1^

The mean age of the patients was 43 years, ranging from 15 to 69 years, i.e., 20 years older than the mean age at which fibroadenoma usually occurs.^1^ The treatment for these lesions is similar to that recommended for carcinomas originating from other parts of the breast. The prognosis is generally good, since the lymph nodes are presented as mostly free from compromise.^1^

The concomitance of fibroadenoma and phyllodes tumor is common, leading some authors to believe that phyllodes tumors originate from previous fibroadenomas.^3^

The malignant phyllodes tumor reported in this paper was a solitary mass of 25 cm with cellular aty- pia, stromatous overgrowth and high mitotic activity (14 mitoses in 10 HPF).

On studying the natural history of fibroadenoma, Carty et al.^3^ reported that the majority of these nodules remain stable or decrease in size. Thus, a passive but watchful conduct for women under 30 years of age is advisable, provided the cytologic or histologic diagnosis of fibroadenoma is confirmed. This will result in mutual benefit for the patient and the health service.

This case report shows two rare situations in a single patient. These demonstrate that although fibroadenomas show self-limited growth, they require periodic clinical control or biopsy, especially when they are detected in women aged over 40 years in whom the frequency of either carcinoma or phyllodes tumors is greater.
